# The Role(s) of Cytokines/Chemokines in Urinary Bladder Inflammation and Dysfunction

**DOI:** 10.1155/2014/120525

**Published:** 2014-03-12

**Authors:** Eric J. Gonzalez, Lauren Arms, Margaret A. Vizzard

**Affiliations:** Department of Neurological Sciences, University of Vermont College of Medicine, D405A Given Research Building, Burlington, VT 05405, USA

## Abstract

Bladder pain syndrome (BPS)/interstitial cystitis (IC) is a chronic pain syndrome characterized by pain, pressure, or discomfort perceived to be bladder related and with at least one urinary symptom. It was recently concluded that 3.3–7.9 million women (>18 years old) in the United States exhibit BPS/IC symptoms. The impact of BPS/IC on quality of life is enormous and the economic burden is significant. Although the etiology and pathogenesis of BPS/IC are unknown, numerous theories including infection, inflammation, autoimmune disorder, toxic urinary agents, urothelial dysfunction, and neurogenic causes have been proposed. Altered visceral sensations from the urinary bladder (i.e., pain at low or moderate bladder filling) that accompany BPS/IC may be mediated by many factors including changes in the properties of peripheral bladder afferent pathways such that bladder afferent neurons respond in an exaggerated manner to normally innocuous stimuli (allodynia). The goals for this review are to describe chemokine/receptor (CXCL12/CXCR4; CCL2/CCR2) signaling and cytokine/receptor (transforming growth factor (TGF-**β**)/TGF-**β** type 1 receptor) signaling that may be valuable LUT targets for pharmacologic therapy to improve urinary bladder function and reduce somatic sensitivity associated with urinary bladder inflammation.

## 1. Lower Urinary Tract (LUT)

### 1.1. Anatomy

The LUT (bladder and urethra) is a division of the renal system that functions to passively store kidney byproducts until it is appropriate to void. To accomplish this, the urinary bladder is a muscular and membranous organ whose structure embodies its reservoir function. Its external features can be organized into an apex, fundus, body, and neck. The apex, or vertex, is on the anterior surface of the urinary bladder and is associated with ligament remnants attached to the umbilicus [1]. The posterior surface is the fundus and its most inferior aspect is termed the base of the urinary bladder [1]. The body typically represents the area between the apex and the fundus and the bladder neck is the most caudal aspect of the inferior bladder surface that is perforated by the internal urethral orifice [1].

The urinary bladder wall is composed of three layers: tunica mucosa, tunica muscularis propria, and tunica serosa/adventitia. The tunica mucosa consists of transitional epithelium and a lamina propria. Transitional epithelial cells in the urinary bladder are termed the urothelium and are arranged in basal, intermediate, and apical cell layers. Basal cells are monolayers directly attached to the basement membrane [2]. Intermediate cells are generally larger in diameter than basal cells and range from one to multiple cell layers depending on the species [2]. The apical, or umbrella, cells are hexagonal in shape and range from 25 to 250 *μ*m depending on urinary bladder distention [2, 3].

Several distinct features of the luminal surface of umbrella cells establish antiadherence and an impermeable barrier characteristic of the urinary bladder mucosa. First, tight junction complexes comprised of occludin and claudin proteins regulate paracellular transport between adjacent umbrella cells [3]. The apical membrane is also occupied by uroplakin, a crystalline plaque cell surface protein that forms an asymmetric unit membrane to maintain impermeability during bladder expansion [4]. Lastly, a layer of proteoglycans on the mucosal surface of umbrella cells serves as an antiadherence factor and provides yet another physical barrier between urinary constituents and the lamina propria [5].

The extracellular matrix of the lamina propria is deep to the basement membrane of the urothelium and contains a diverse array of interstitial cells, nerve terminals, and vasculature [3, 6]. It has been suggested that the lamina propria may have an important role in integrating epithelial and smooth muscle function due to its innervations and proximity to the urothelium and tunica muscularis propria [6]. The tunica muscularis propria consists of three smooth muscle layers termed the detrusor. The internal and external layers are arranged longitudinally, whereas those in the middle are circular [7, 8]. The smooth muscle cells in the muscularis propria retain their classic spindle shape and are bundled together by collagen-rich connective tissue [7]. External to the muscularis propria, the tunica serosa surrounds the superior and lateral surfaces of the urinary bladder wall, whereas the retroperitoneal aspects contain a vascular, loose connective tissue termed the tunica adventitia [8].

Caudal to the inferior surface of the urinary bladder is the urethra. Similar to the urinary bladder wall, the urethral wall is composed of a tunica mucosa, tunica muscularis propria, and tunica adventitia. The tunica mucosa consists of transitional epithelium proximal to the urinary bladder followed by nonkeratinized, stratified squamous epithelium distally [8, 9]. The tunica muscularis propria is composed of inner and outer smooth muscle arranged longitudinally and circularly, respectively [8]. In the male urethra, the circular smooth muscle fascicles join with urinary bladder smooth muscle at the urethrovesical junction to form the internal urethral sphincter [8, 9]. The smooth muscle fascicles along the proximal female urethra, however, do not appear to anatomically arrange into a sphincter [9]. Skeletal muscle of the urethral wall forms the external urethral sphincter and extends along the membranous urethra in males to generate voluntary pressure during bladder filling [10]. The skeletal muscle fibers in the female urethra join to form an “external” urethral sphincter comprised of a sphincter urethrae, compressor urethrae and sphincter urethrovaginalis to provide urinary continence through urethral and vaginal closure [11].

### 1.2. Neural Control

The LUT is regulated by supraspinal, spinal, and peripheral nervous system (PNS) input to maintain “switch-like” patterns of storage and elimination activity and has been previously reviewed in greater detail [10]. Briefly, bladder wall mechanoreceptors initiate visceral afferent (A*δ* fibers) activity during the storage phase that synapse on spinal interneurons [10, 12]. Spinal reflex pathways then facilitate storage by directly enhancing thoracolumbar sympathetic outflow and somatomotor discharge or ascending, in some species, to keep metencephalic integration centers [10, 12].

Spinal interneurons activate preganglionic sympathetic fibers from the intermediolateral cell column of the lower thoracic (T10) through upper lumbar (L2) spinal cord that form thoracic and lumbar splanchnic nerves [13, 14]. The preganglionic fibers then synapse on the prevertebral inferior mesenteric ganglia or paravertebral ganglia and travel along the hypogastric and pelvic nerves, respectively [10]. Adrenergic neurotransmission on the urinary bladder smooth muscle *β*-adrenergic receptors promotes bladder wall relaxation and accommodation [13]. Bladder filling is also facilitated by the activation of *α*-adrenergic receptors on the internal urethral sphincter resulting in contraction of the urethral outlet [13]. Spinal reflex pathways not only enhance sympathetic outflow but also *α*-motoneuron discharge from Onuf's nucleus in the ventrolateral horn of the sacral (S2–S4) spinal cord [12]. Propagation of this signal along the pudendal nerve to the external urethral sphincter elicits skeletal muscle contraction by activating nicotinic acetylcholine receptors to provide voluntary control over urinary continence [13].

Upon reaching the tension threshold, bladder afferents (A*δ* fibers) bypass local spinal reflexes and ascend to the mesencephalic periaqueductal gray (PAG). Unlike the reflexes underlying the storage phase, the elimination phase relies on supraspinal circuitry as evidenced by voiding dysfunction following lower thoracic spinal cord injury [14, 15]. After cortical processing, the PAG sends excitatory input to a region in the dorsolateral pontine tegmentum termed the pontine micturition center (PMC) [16]. The PMC then sends descending cortical projections that synapse on preganglionic parasympathetic neurons and inhibitory interneurons in the sacral spinal cord [14, 16].

The preganglionic parasympathetic fibers arise from the intermediolateral cell column of the sacral (S2–S4) spinal cord to form pelvic splanchnic nerves. Upon coursing through and exiting the hypogastric and pelvic plexus, the fibers join the pelvic and pudendal nerves to synapse on terminal ganglia and innervate the detrusor smooth muscle and urethra [12, 13]. Cholinergic and nonadrenergic/noncholinergic neurotransmission on the urinary bladder smooth muscle promotes bladder wall contraction by activating muscarinic acetylcholine receptors and purinergic receptors, respectively [14]. Elimination of urine is also facilitated by nitric oxide release onto the internal urethral sphincter resulting in a relaxation of the urethral outlet [14]. The PMC not only augments parasympathetic outflow but also attenuates preganglionic sympathetic and *α*-motoneuron discharge to the LUT [16]. The descending cortical projections terminating on inhibitory interneurons in the sacral spinal cord prevent excitatory input into the urethral sphincters resulting in dilation of the urethral orifice and continuous flow of urine. As distention of the urinary bladder decreases during the elimination phase, ascending excitation to the dorsolateral metencephalon is diminished and the storage phase is once again switched on.

### 1.3. Symptoms and Dysfunction

The terminology used in the following section is consistent with the standardization report of LUT symptoms and function by the International Continence Society and will refer to their definitions when appropriate [17]. Similar to other clinical indications, LUT symptoms are the patient's qualitative representation of a purported condition. These symptoms, in particular, refer to a spectrum of LUT functions that include storage, elimination, and postmicturition disturbances.

Symptoms associated with the storage phase include, but are not limited to, “increased frequency, urgency, and incontinence” [17]. The complaint of increased urinary frequency is prevalent among both men and women with LUT dysfunction and has been suggested to affect an individual's quality of life as demonstrated by a strong correlation between frequency and bothersome endorsements [18]. Increased urgency is a complaint of the “sudden compelling desire to pass urine” that may be accompanied by pain, pressure, or discomfort associated with the LUT [17]. Lastly, urinary incontinence includes a complaint of the “involuntary leakage of urine” and may manifest in various forms and severities [17]. It is important to note that incontinence is not representative of one particular LUT dysfunction but rather can arise from multiple sources including stress, comorbid disorders, and congenital abnormalities [19].

Symptoms associated with the elimination phase include “hesitancy, slow or intermittent stream, straining, and terminal dribble” [17]. These symptoms generally involve complaints of the initiation and continuation of voiding and alterations to their urine stream and appear to be more prevalent in men compared to women [17, 18]. Symptoms associated with the postmicturition phase occur after voiding and include “incomplete emptying and postmicturition dribble” [17]. Although equally bothersome, postmicturition dribble may be more prevalent in men, whereas, in women, incomplete emptying may be more prevalent [18]. As briefly mentioned above, LUT symptoms are not confined to urodynamic disturbances but may also include unpleasant sensations of pain or discomfort during storage or elimination. These sensations are generally perceived to emanate from the urogenital organs and may exacerbate storage and elimination symptoms [20].

## 2. Bladder Pain Syndrome (BPS)/Interstitial Cystitis (IC)

### 2.1. Background

LUT signs and symptoms resembling what is currently termed BPS/IC have been documented throughout history and its perspective has been previously reviewed in detail [21]. Briefly, Drs. Philip Syng Physick and Joseph Parish first recognized an inflammatory condition called tic douloureux of the bladder whose symptoms included chronic urinary frequency, urgency, and pelvic pain [22]. Skene [23] expanded the cystoscopic features of this concept in the late 19th century and introduced the term IC which included ulceration of the mucous membrane and inflammation within the bladder wall. Focal, ulcerative bleeding in the urinary bladder wall remained a hallmark of IC due, in part, to the work of Hunner [24] in the early 20th century [21]. Many patients, however, were misdiagnosed as current estimates suggest only 5–7% of those with BPS/IC present with bladder ulcerations [21, 25].

In the absence of a formal classification for IC, the National Institute of Diabetes and Digestive and Kidney Diseases (NIDDK) attempted to standardize its research definition in 1987 by establishing a diagnostic criteria [26]. The criteria included the presence of “glomerulations on cystoscopic examination or a classic Hunner ulcer, pain associated with the bladder, urinary urgency” and eighteen exclusion conditions [21, 26]. After several iterations and international consultations, the term IC was expanded to include BPS [27, 28]. The patient selection for BPS is based on “chronic pelvic pain, pressure, or discomfort perceived to be related to the urinary bladder accompanied by at least one other urinary symptom such as persistent urge to void or frequency,” whereas IC is “reserved for cystoscopic and histological features” [17, 28]. At this time, the terms BPS and BPS/IC are analogous and are defined by the American Urological Association Interstitial Cystitis Guidelines Panel as at least six weeks of LUT symptoms and unpleasant sensations perceived to be related to the urinary bladder and with no other clinically identifiable sources [27].

### 2.2. Epidemiology

The epidemiology of BPS/IC is limited due to the absence of standardized definitions, markers, and examinations [21, 25, 29, 30]. Taking into account this variability, it is estimated that there is a 5 : 1 female-to-male ratio among BPS/IC patients [21, 25]. It is estimated that 300 per 100,000 women worldwide suffer from BPS/IC [21]. In the United States alone, 3.3 to 7.9 million women are estimated to meet the criteria for BPS/IC [30]. As expected, BPS/IC puts an enormous financial burden on the individual and economy as a whole. Health care costs for an individual with BPS/IC range from 4 to 7 thousand dollars per year, while the economic burden approaches 500 million dollars per year in lost productivity and therapeutics [21, 31].

### 2.3. Pathophysiology

**Figure 1:**
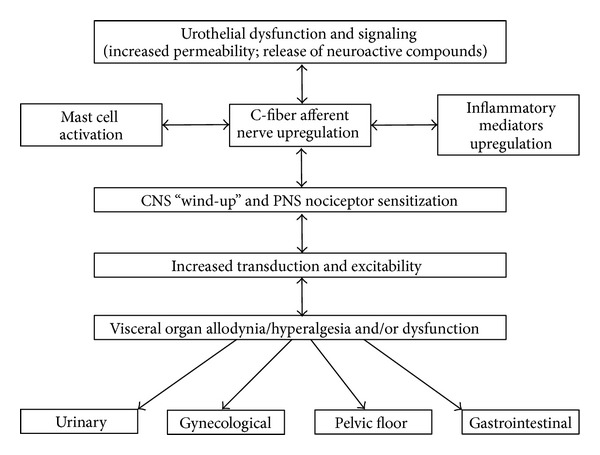
Potential etiologic cascade and pathogenesis underlying painful bladder syndrome (BPS)/interstitial cystitis (IC). It is likely that BPS/IC has a multifactorial etiology that may act predominantly through one or more pathways resulting in the typical symptom-complex. There is a lack of consensus regarding the etiology or pathogenesis of BPS/IC but a number of proposals include a “leaky epithelium,” release of neuroactive compounds at the level of the urinary bladder with mast cell activation, “awakening” of C-fiber bladder afferents, and upregulation of inflammatory mediators including cytokines and chemotactic cytokines (chemokines). Inflammatory mediators can affect CNS and PNS neural circuitry including central “wind-up” and nociceptor sensitization resulting in chronic bladder pain and voiding dysfunction. BPS/IC is associated with diseases affecting other viscera and pelvic floors. See text for additional details. Figure adapted from [32].

While the primary insult underlying BPS/IC is not known, it has been suggested that the pathophysiology is a “vicious circle” involving uroepithelial dysfunction, inflammation, afferent nerve hyperexcitability, and visceral hyperalgesia and allodynia (Figure 1) [32]. This section will explore the said mechanisms that have been proposed to feedforward to promote the chronicity of LUT symptoms observed in BPS/IC [32].

The urothelium is a specialized, stratified epithelium that when intact provides a nonadherent, passive barrier through tight junction proteins, plaque proteins, and surface proteoglycans [2]. Any perturbation to the components of this permeability barrier may lead to increased infiltration into the bladder wall and exposure of the interstitium to urinary constituents [33–36]. The diffusion of urinary constituents like potassium into the bladder interstitium may depolarize muscle and nerve cells, inflame tissues, degranulate mast cells, and cascade to the development of LUT symptoms (Figure 1) [35]. Uroepithelial dysfunction specific to BPS/IC, however, remains controversial. For example, Chelsky et al. [37] demonstrated that the permeability in IC was comparable to the variation seen in symptom-free controls, whereas Parsons et al. [36] demonstrated abnormal permeability and potassium absorption in those with IC [21]. The abundance of studies for or against uroepithelial dysfunction in BPS/IC suggests that it may not be a primary insult but rather may occur in a subset of patients to exacerbate LUT symptoms [21].

In addition to the uroepithelial disruption, visceral inflammation also remains a central pathological process in BPS/IC and has been suggested to underlie the development of LUT symptoms (Figure 1). Inflammation within the urinary bladder viscera is characterized by increased vasculature, mucosal irritation that may result in barrier dysfunction, and infiltration of inflammatory mediators [38, 39]. The proliferation and activation of mast cells, in particular, have received considerable attention in the urinary bladder immune response [32]. Mast cells secrete vasoactive chemicals to promote innate and autoimmunity and their increased activity has been widely demonstrated in BPS/IC [40–43]. The subsequent exposure in the bladder interstitium to vasoactive chemicals, inflammatory mediators, and neuropeptides from visceral inflammation may lead to afferent nerve hyperexcitability and neurogenic inflammation (Figure 1) [44–46].

The loss of inhibition on peripheral afferents (A*δ* and C fibers) increases input into the spinal cord and may eventually promote central sensitization [32]. An unregulated state of central and peripheral reactivity causes “wind-up” which is observed clinically as hyperalgesia and allodynia (Figure 1). In BPS/IC, hyperalgesia and allodynia are characterized by an elevated state of urinary bladder sensation that may cause pain, pressure, or discomfort and may result in increased urinary frequency and urgency [38]. The “vicious circle” continues as mast cell degranulation and infiltration of mediators from uroepithelial dysfunction and/or visceral inflammation sustain peripheral and central sensitization to establish visceral hyperalgesia/allodynia and chronic LUT symptoms (Figure 1) [32].

### 2.4. Animal Models

Numerous animal models have been implemented to determine the onset and chronicity of LUT dysfunctions like BPS/IC. While one model cannot currently account for the constellation of symptoms in BPS/IC, they each aid in identifying distinct mechanisms underlying part of its pathophysiology. This section will explore a naturally occurring cystitis model in felines and focus its review on experimental models of cystitis induced chemically. It is important to note that models of BPS/IC are not limited to what will be discussed in this section and exhaustive reviews have been previously published [47–49].

The natural development of spontaneous LUT symptoms has been documented in cats for several decades and is termed feline interstitial cystitis (FIC) [47, 50]. Though the primary insult for FIC is not known, the pathophysiology has marked similarities to BPS/IC including uroepithelial dysfunction and visceral inflammation. Cats with FIC have been shown to have a disruption to the epithelial cytoarchitecture that increased diffusion and infiltration of urinary constituents [51, 52]. Uroepithelial dysfunction in FIC further led to a peripheral upregulation of neuropeptides and inflammatory mediators that altered bladder afferent soma size and increased input to the central nervous system (CNS) [53]. As previously discussed, the alterations to central and peripheral reactivity following uroepithelial dysfunction and/or visceral inflammation may promote the development of LUT symptoms that is observed in FIC and, by extension, BPS/IC [32, 38, 53].

Despite these pathophysiological similarities, FIC as a model for BPS/IC is limited due to its spontaneity and epidemiology. Investigators are practically and financially restricted to structural and functional alterations following its spontaneous induction and thus inadequately define insults preceding the development of FIC [49]. Furthermore, unlike BPS/IC, FIC occurs irrespective of biological sex [49]. While this may be due to a misdiagnosis of BPS/IC in males, one cannot discount hormonal differences that may affect LUT symptoms in humans [54, 55].

LUT symptoms have also been induced by an assortment of chemical irritants including, but not limited to, hydrochloric acid, acetic acid, protamine sulfate (PS), and cyclophosphamide (CYP). The inflammation induced by intravesical instillation of irritants like hydrochloric acid and acetic acid helps reveal the anatomical, organizational, and functional alterations attributable to the visceral immune response [47]. Specifically, the functional and histological features following acid instillation are similar to a BPS/IC subset and include urothelial hyperplasia, bladder ulceration, mucosal edema, inflammatory cell infiltration, and the development of LUT symptoms [56, 57]. Though acid instillation allows for a more controlled environment than FIC, the studies must be interpreted cautiously as the degree of inflammation resulting from exogenous irritants may not be representative of the naturally occurring BPS/IC [47].

Unlike acid instillation, PS lacks a pervasive inflammatory element but rather disrupts uroepithelial barrier function by targeting bladder surface proteoglycans [58]. Similar to the uroepithelial dysfunction observed in FIC, PS instillation is sufficient to induce LUT symptoms [59]. More recently, PS has been used in conjunction with bacterial induced cystitis. Instillation of both PS and* E. coli* lipopolysaccharide to, respectively, damage the urothelium and induce a visceral inflammatory cascade may help clarify the interaction(s) of multiple processes underlying LUT symptoms in BPS/IC [47, 60].

CYP is an antineoplastic prodrug that requires enzymatic activation to release phosphoramide mustard and the byproduct acrolein [61, 62]. A known adverse toxicity following systemic CYP administration is hemorrhagic cystitis [62]. Hemorrhagic cystitis is considered to arise from the bladder mucosal walls contact with acrolein, which has been shown to increase vascular permeability and result in bladder ulceration and hypertrophy [63]. In addition to hemorrhagic cystitis, systemic CYP treatment causes functional and histological changes similar to BPS/IC including mucosal edema, uroepithelial dysfunction, inflammatory cell infiltration, afferent nerve hyperexcitability, and the development of LUT symptoms [45, 64–67]. CYP administration also produces behavioral alterations consistent with the development of viscerosomatic pain including decreased breathing rate, closing of the eyes, and rounded back postures [66]. While the urinary bladder inflammatory response following systemic CYP administration is greater than what is observed in BPS/IC, this experimental model of cystitis is appealing because of its route of administration (intraperitoneal) and the chronicity and reproducibility of histopathological and functional alterations [47].

## 3. Inflammatory Mediators in Urinary Bladder Inflammation

We have hypothesized that pain associated with BPS/IC involves an alteration of visceral sensation/bladder sensory physiology. Altered visceral sensations from the urinary bladder (i.e., pain at low or moderate bladder filling) that accompany BPS/IC [68–72] may be mediated by many factors including changes in the properties of peripheral bladder afferent pathways such that bladder afferent neurons respond in an exaggerated manner to normally innocuous stimuli (allodynia). These changes may be mediated, in part, by inflammatory changes in the urinary bladder (Figure 1). Among potential mediators of inflammation, neurotrophins (e.g., nerve growth factor, NGF) have been implicated in the peripheral sensitization of nociceptors [73–75]. Proinflammatory cytokines also cause sensitization of polymodal C-fibers [74] and facilitate A-beta input to the spinal cord [76, 77]. Several studies from our laboratory have demonstrated increased expression of cytokines and chemokines (chemotactic cytokines) and the beneficial effects of receptor blockade in the urinary bladder after CYP-induced bladder inflammation [64]. In the next sections, we will present a summary of recent studies from our laboratory that addresses the role(s) of two chemokine/receptor pairs (CXCL12/CXCR4; CCL2/CCR2) and the cytokine/receptor pair (transforming growth factor (TGF-*β*)/TGF-*β* type 1 receptor) in urinary bladder inflammation and somatic sensitivity in a CYP rat model of urinary bladder inflammation.

Using the CYP-induced bladder inflammation model, we aimed to characterize further the role of inflammatory chemicals in the development and/or maintenance of neuronal sensitization and chronic pain states associated with BPS/IC. Inflammatory chemicals are released at sites of injury and inflammation by resident and infiltrating immune cells and endothelial and parenchymal cells. Proinflammatory molecules act to heal the injured/inflamed area and also to sensitize nociceptive neurons, thus increasing the pain response in order to prevent further insult [78]. While initial immune activation and sensitization of sensory neurons is protective, prolonged inflammatory processes and sensory sensitization occurring after tissue healing are associated with chronic pain syndromes, including BPS/IC. Various cytokines and chemokines have been detected in the urine and urinary bladder in models of cystitis and patients with BPS/IC and therefore may represent novel therapeutic targets or biomarkers for the syndrome.

## 4. Chemokines

### 4.1. Background

Chemokines are a large family of structurally and functionally related proteins that are important mediators of immune responses, inflammatory processes, and nociception. In the immune response, chemokines facilitate tissue recovery by causing the extravasation of leukocytes from blood plasma to the site of injury. Chemokine receptors present on leukocytes sense increasing chemotactic concentration gradients and facilitate cellular motility towards them [79].

Chemokines are small, secreted proteins of approximately 100 amino acids in length that comprise 4 subfamilies: CC, CXC, CX3C and C (review see [80]). Each subfamily is named for the first cysteine residue motif from its amino terminus. Families of chemokines assert their actions by signaling via related G-coupled protein receptors. Within each subfamily, receptor and ligand pairing is not mutually exclusive; in other words, multiple ligands can bind the same receptor and vice versa (for review see [80]). The complexity of chemokine receptor binding presents challenges when examining the functional role of chemokine signaling. Despite difficulties, determining the role of chemokines and their receptors in both control and pathological states could provide insights to possible therapeutic interventions in a variety of chronic pain conditions including BPS/IC.

### 4.2. Chemokines and Peripheral Sensitization

Inflammatory mediators such as proinflammatory cytokines (e.g., tumor necrosis- (TNF-)*α*, interleukin- (IL-)6, IL-1*β*), COX-2, NGF, protons, prostaglandins, and bradykinin have been implicated in the direct sensitization of nociceptive afferents [81]. Traditionally, chemokines were not thought to assert direct effects on primary sensory neurons. Rather, chemokine/receptor interaction on the plasma membrane of leukocytes was thought to stimulate leukocyte release of nociceptive mediators via GCPR signaling mechanisms [78]. However, electrophysiological, expressional, and functional pain studies have demonstrated the possibility of direct chemokine-mediated neuronal hypersensitivity and pain. For example,* in vitro*, exogenous chemokine application can physiologically alter sensory neurons by changing membrane potentials [82], decreasing thresholds for action potential generation [82], increasing excitability, and evoking discharges [82, 83]. Various chemokines, including CXCL12, can modulate calcium ion currents in cultured DRG cells, potentially facilitating hyperexcitability [84–87]. In a neuronal injury model, chronic compression of the DRG elicits a depolarizing response to chemokines that was not detected in control (noncompressed) DRG [83]. Chemokine-mediated sensitization may involve members of the transient receptor potential family, including TRPV1. Various chemokines and receptors colocalize with neuronal TRPV1 as well as neuropeptides released in a TRPV1-dependent manner [87–90].

Following nerve injury or inflammation, expression of chemokines and associated receptors increases significantly in macrophages, infiltrating T cells, sensory neurons, and glia [79, 84, 85, 89, 91–93]. Additionally, increased neuronal activity, as would occur during injury or inflammation, has been shown to induce chemokine transcription in cultured DRG neurons [94]. Cytokines such as IL-1 can increase chemokine expression in neurons and astrocytes [95–97]. Chemokine expression in, and subsequent secretion from the various cell types (e.g., leukocytes, endothelial cells, neurons, or parenchymal cells) would enable chemokines, by diffusion, to interact with functional chemokine receptors on DRG neurons thus facilitating hyperexcitability changes such as those described above.

### 4.3. Chemokines and Central Sensitization

Increased primary afferent signaling can induce organizational and neurochemical changes in spinal cord synapses that underlie the phenomenon, central sensitization, which may contribute to chronic pain syndromes. During central sensitization, intensely heightened peripheral input decreases thresholds necessary to elicit action potentials in dorsal horn neurons. An increase in nociceptive neurotransmitter (e.g., SP and CGRP) release into the dorsal horn could increase the activity of spinal neurons that mediate both local reflexes and ascend to higher brain centers, thus facilitating the perception of pain [78, 87, 98].

Chemokine/receptor signaling may contribute to central sensitization via activation of either the peripheral or central afferent limbs of pain pathways [78, 98]. Chemokine activation of peripheral DRG neurons was described in the previous section. Additionally, chemokine application can evoke SP and CGRP release from DRG neurons that could cause chemokine-mediated central effects indirectly [87, 99]. Evidence suggests that chemokines may have direct central effects also. Jung et al. [89] detected large dense-core vesicles containing both CCL2 and CGRP in TRPV1-expressing DRG neurons. Dansereau et al. [88] demonstrated calcium-evoked release of CCL2 following incubation of DRG neurons with potassium or capsaicin. CCL2 may traffic anterogradely from the soma of peripheral sensory neurons and is increased in the supernatant following intense stimulation of mechanically injured DRG neurons [100]. Additionally, CCL2 application increases the frequency of spontaneous EPSCs in superficial dorsal horn neurons [101]. Chemokines released from primary afferent central terminals could exert either direct activation of superficial dorsal horn neurons via functional chemokine receptor expression or indirect sensitization via activation of microglia and astrocytes that subsequently release nociceptive mediators.

Chemokine receptors, including CCR2, and chemokines are detected in dorsal horn neurons and activated astrocytes and microglia in numerous models of neuropathic pain including peripheral or central nerve damage or tissue inflammation [102–107]. Chemokine cross signaling between bladder sensory afferents and microglia or astrocytes could modulate symptoms of BPS/IC especially considering that peripheral injury or inflammation (e.g., bladder) can induce central glial activation.

### 4.4. Chemokines and Nociception

Studies investigating nociceptive behavior illustrate a strong relationship between chemokines and pain. Exogenous administration of chemokines induces thermal hyperalgesia and mechanical allodynia [85, 87, 93] while certain chemokine knockout mice fail to develop somatic sensitivity [108, 109]. Oh and colleagues [87] published an early example of nociceptive chemokine function when they showed that intraplantar administration of various chemokines such as CXCL12, CCL5, and CCL22 induce mechanical hypersensitivity lasting for at least 3 h. Since then numerous studies have reported that either exogenous peripheral (e.g., intradermal) or central (e.g., intrathecal) chemokine application induces mechanical hypersensitivity and/or thermal hyperalgesia [88, 90, 92, 99, 110, 111]. Intrathecal administration of CCL2 can produce mechanical hypersensitivity within 30 minutes and hyperalgesic effects can be detected up to 4 days after administration [88]. In contrast, CCL2-induced thermal hypersensitivity resolves 24 h after administration [88]. Interestingly, transgenic mice lacking the CCR2 receptor (principle receptor for CCL2) are resistant to the development of mechanical hypersensitivity following mechanical nerve injury; however, following complete Freund's adjuvant- (CFA-) induced neural inflammation, these mice display only a small, insignificant decrease in mechanical sensitivity compared to control animals and show no changes in thermal nociception [108]. These data suggest specificity for chemokine function with respect to type of injury and pain modality.

Studies utilizing antagonists against chemokine signaling provide evidence for a therapeutic role with respect to neuropathic pain. In two different models of HIV-1 associated neuropathy, Bhangoo et al. [85, 91] demonstrate that antiretroviral drug- or viral coat protein-, gp120-, induced mechanical hypersensitivity is attenuated by acute, systemic treatment with CCR2 or CXCR4 antagonists. Other pain eliciting models such as focal demyelination, CFA-induced inflammation and sciatic nerve constriction have demonstrated the therapeutic effects of chemokine receptor antagonists [84, 111, 112].

### 4.5. Chemokines and Cystitis

Clinical studies assessing patients with various pelvic inflammatory/pain syndromes and rodent models of visceral inflammation indicate a role for chemokines in the initiation or maintenance of visceral inflammation. CYP-induced inflammation increases the expression of CXCL12/CXCR4, CX3CL1/CX3CR1, CCL2/CCR2, and CXCL1 in the urinary bladder and CCL2 and CXCL1 in urine [113–117]. Blockade of CXCL10 signaling reduces severity of CYP-induced bladder inflammation by reducing hyperplasia, epithelial erosions, and infiltration of T cells, mast cells, and killer T cells in the bladder urothelium of rats [118]. Additionally, elevated chemokines levels have been detected in the seminal plasma and peripheral immune cells of patients with pelvic inflammatory/pain syndromes such as ulcerative colitis, chronic prostatitis, chronic pelvic pain syndrome, and BPS/IC [118–120]. Bladders from patients with ulcerative BPS/IC have increased mRNA expression of CXCL9, CXCL10, and CXCL11 in the interstitium and CXCR3 in the urothelial membrane [121]. Both Tyagi et al. [122] and Corcoran et al. [123] detected elevated chemokines, specifically CXCL1, CXCL10, CXCL12-*α*, and CCL7, in the urine of patients with ulcerative BPS/IC. Interestingly, CCL7 levels decreased following hydrostatic distention and were correlated with symptom relief [123]. Tyagi et al. [122] suggest the presence of urinary chemokines originates from bladder tissue because urinary CXCL10 levels are present at levels much higher than those detected in serum.

Considering the extensive data implicating a sensory and signaling role for the urothelium, it is possible that urothelial-derived chemokines, especially those detected in the urine of BPS/IC patients, contribute to symptoms of bladder dysfunction. Recently, the functional contribution of the urothelium has advanced beyond the view of a passive barrier and is now suggested to have “neuron-like” properties such as plasticity and sensory transduction capabilities, especially in the context of bladder inflammation [124, 125]. Functional receptor expression, in conjunction with secretion capabilities, allows the urothelium to respond to stimuli and reciprocally communicate with detrusor smooth muscle cells, suburothelial nerve plexus, or interstitial cells [126–130]. It is possible that chemokine signaling via receptor expression in urothelial cells may consequently activate downstream targets that promote either the transcription or the expression and release of other inflammatory mediators or excitatory amino acids. Urothelial derived mediators such as adenosine triphosphate or nitric oxide may then influence the suburothelial nerve plexus to affect micturition reflex function [129].

## 5. CXCL12 and CXCR4 

Our lab examined the expression and therapeutic effect with receptor blockade of the chemokine CXCL12, and one of its two receptors, CXCR4, in a rodent model of cystitis. This chemokine/receptor pair was of interest because of its demonstrated role in visceral inflammation and pathology in other abdominopelvic organs. Mikami et al. [119] show that CXCR4 peripheral T-cell expression was increased in patients with ulcerative colitis and that expression levels correlated with disease activity. Additionally, chemically induced colitis in mice leads to an increase of CXCR4-positive leukocytes and CXCL12 expression in colonic tissue [119]. Administration of a CXCR4 antagonist reduced these inflammatory effects. To address the role of CXCL12/CXCR4 signaling in normal micturition and inflammation-induced bladder hyperreflexia, bladder inflammation in adult female Wistar rats was induced by injecting CYP intraperitoneally at acute (150 mg/kg; 4 h), intermediate (150 mg/kg; 48 h), and chronic (75 mg/kg; every third day for 10 days) time points. CXCL12 and its receptor, CXCR4, were examined in the whole urinary bladder of control and CYP-treated rats using complementary approaches including enzyme-linked immunosorbent assays (ELISAs), qRT-PCR, and immunostaining techniques. ELISAs, qRT-PCR, and immunostaining experiments revealed a significant increase in CXCL12 and CXCR4 expression in the whole urinary bladder and particularly in the urothelium, with CYP treatment [114]. CXCL12/CXCR4 interactions in micturition were evaluated using conscious cystometry with continuous instillation of saline and CXCR4 receptor antagonist (AMD3100; 5 *μ*M) administration in control and CYP- (48 h) treated rats. Receptor blockade of CXCR4 using AMD3100 increased bladder capacity in control (no CYP) rats and reduced CYP-induced bladder hyperexcitability as demonstrated by significant increases in intercontraction interval, bladder capacity, and void volume [114]. In these studies, AMD3100 is most likely acting at the level of the urothelium for several reasons: (1) both mRNA and histologic analyses showed that the greatest expressional increase for both CXCL12 and CXCR4 following CYP treatment was in the urothelium; (2) histologically, CXCR4 had a restricted presentation being expressed only in the urothelium in both control and CYP treated bladders; (3) repeated attempts did not demonstrate CXCL12- or CXCR4-IR in the suburothelial nerve plexus [114]. These results suggest a role for CXCL12/CXCR4 signaling in both normal micturition and with bladder hyperreflexia following bladder inflammation.

## 6. CCL2/CCR2

The chemokine, CCL2 (monocyte chemoattractant protein-1, MCP), and its high-affinity receptor, chemokine (C–C motif) receptor 2 (CCR2), have been implicated in hypersensitivity following neuronal inflammation or mechanical injury [88, 92, 99, 101, 108, 131, 132] in the central (i.e., spinal cord) and peripheral (i.e., DRG) nervous system. Blockade of CCR2 reduces established pain behaviors resulting from chronic nerve injury [88, 99, 101, 132] and exogenous application of CCL2, either centrally or peripherally, can elicit exaggerated sensory behavioral responses in rodents [88, 92, 99, 101, 132]. In addition, CCR2 null mice fail to develop somatic sensitivity following partial sciatic nerve ligation [108] whereas mice with CCL2 overexpression in astrocytes develop exaggerated thermal hyperalgesia following complete Freund's adjuvant-induced inflammation [131].

Our recent studies demonstrate novel findings with respect to the contribution of CCL2/CCR2 interactions with bladder inflammation-induced changes in bladder function and somatic sensitivity in female rats. We demonstrate that CYP-induced cystitis increases (1) CCL2 and CCR2 transcript and protein expression in the rat urinary bladder and (2) the number of bladder-associated CCR2-immunoreactive bladder afferent cells in the lumbosacral DRG [113]. Blockade of CCR2 receptor interactions with the highly selective receptor antagonist, RS504393 (5 *μ*M), at the level of the urinary bladder, increased bladder capacity, decreased void frequency, and reduced somatic sensitivity of the hindpaw and pelvic region following CYP treatment [113]. These results extend previous findings [83, 88, 92, 133, 134] by demonstrating that CCL2/CCR2 interactions contribute to inflammation-induced bladder dysfunction and increased referred somatic sensitivity.

CCL2/CCR2 interactions at the level of the urothelium and suburothelial nerve plexus in the urinary bladder are likely to contribute to bladder dysfunction and increased somatic sensitivity following CYP-induced cystitis. Intravesical instillation of RS504393 likely makes direct contact with the urothelium that expresses CCR2 and the increased urothelial permeability due to CYP treatment makes it likely that intravesical RS504393 also contacts suburothelial nerves. Our studies did not differentiate between direct urothelial and nerve-mediated CCR2 effects versus indirect urothelial-mediated communication with the detrusor smooth muscle, suburothelial nerve plexus, and/or interstitial cells as previously suggested [126, 127]. It is possible that urothelial CCL2/CCR2 signaling facilitates the release of urothelial-derived mediators such as adenosine triphosphate or nitric oxide that may then influence underlying structures such as the suburothelial nerve plexus and/or detrusor smooth muscle [126, 127, 129].

Alternatively, or in addition to urothelial-mediated mechanisms, CCL2/CCR2 interactions in bladder associated DRG neurons may contribute to inflammatory-induced changes in bladder sensory physiology and function. CYP treatment triggered a robust increase in the percentage of bladder afferent cell bodies expressing CCR2-IR [113]. These results complement previous findings demonstrating an increase in the percentage of primary sensory afferent cells expressing CCL2 and/or CCR2 following focal nerve demyelination, sciatic nerve ligation, or chronic constriction injury [83, 84, 89, 92, 133, 135]. Jung and Miller [94] demonstrate that depolarization of cultured sensory neurons is sufficient to induce CCR2 mRNA expression suggesting that heightened sensory neuron activity during states of injury or inflammation may contribute to elevated levels of neuronal CCR2 expression. Increased receptor expression may explain why peripheral nerve damage or inflammation can also change the functional properties of sensory neuron populations such that an increasing percentage of DRG neurons responds to CCL2 application or neurons respond with increased intracellular calcium ion currents and/or frequency of EPSCs [82–84, 99, 101, 135]. Therefore, it is possible that CCL2 released,* in vivo*, by DRG neurons, glial cells, and urothelial cells could contribute to nociceptive sensations/behaviors by autocrine or paracrine signaling mechanisms.

## 7. Cytokines

In addition to the chemokine family, ample evidence suggests that other cytokines contribute to the development of hyperalgesia and allodynia following injury or inflammation [79, 136]. Cytokine receptors have been detected in neurons and glial cells, especially after peripheral neuropathy [136]. Cytokine/receptor interactions can activate signaling pathways that induce transcription and release of other proinflammatory/nociceptive mediators including NGF and other cytokines and chemokines from peripheral neurons or glial cells [95–97, 137]. Cultured human detrusor smooth muscle cells secrete low levels of cytokines (IL-6 and IL-8) and chemokines (CCL2 and CCL5) and exposure to the inflammatory cytokines, IL-1*β* and TNF-*α*, increases this release [138, 139]. The expression of cytokines, alone or in combination with other cytokines, growth factors, or other mediators, may form a bidirectional communication network between the nervous system and the immune system [140].

Studies examining cytokine expression using a CYP model of cystitis have detected elevated IL-6, IL-1*α*, and IL-4, among others, protein and mRNA levels in the urine and urinary bladder [64, 115]. Cytokine transcription and expression increase in the urinary bladder of patients with ulcerative BPS/IC [121, 123, 141]. Certain cytokine mRNAs, including IL-6 and TNF-*α*, have been detected in the interstitium and urothelium of these biopsies [121, 141]. Additionally, reports have repeatedly detected elevated IL-6 in the urine of patients with ulcerative BPS/IC. Increased levels have been suggested to indicate either severity of inflammation [39] or correlate with pain scores and nocturia [141, 142]. Lotz et al. [141] propose that the bladder is the primary source of urinary IL-6 because it was not detected in ureteral urine.

Recently we examined the expression and function of another cytokine, TGF-*β*, in the urinary bladder with inflammation. TGF-*β* has an extensive role in the immune system and has been implicated in nociception and detected in the urine and urothelium of rats treated with CYP-induced cystitis [143].

## 8. Transforming Growth Factor-Beta (TGF-**β**)

### 8.1. Background

The TGF-*β* superfamily is comprised of at least 35 pleiotropic proteins belonging to four subfamilies grouped by their sequence homology—decapentaplegic-Vg-related (DVR), activin/inhibin, TGF-*β sensu stricto,* and other divergent members [144]. Even though TGF-*β* superfamily members have distinct expression patterns and regulate a variety of functions, they are each translated as a preproprotein that contains a peptide sequence signaling to the endoplasmic reticulum, a N-terminal prodomain, and a C-terminal mature protein [144, 145]. After proteolytic processing and posttranslational modifications, the C-terminal fragment is either secreted as a mature protein dimer or forms a latent complex by maintaining a noncovalent bond to the prodomain [144, 145].

The canonical members of TGF-*β sensu stricto *are one such proprotein to form a latent complex. The interactions between the N-terminal prodomain, termed latency associated peptide (LAP), and the mature TGF-*β* dimer are sufficient to sequester its extracellular activity [146]. Additionally, LAP associates with a latent TGF-*β* binding protein (LTBP) that regulates TGF-*β* bioavailability by chaperoning the complex to the extracellular matrix [147]. The subsequent activation of latent TGF-*β* in the extracellular matrix via LAP cleavage occurs by protease-dependent or protease-independent (protons, integrins, reactive oxygen species, etc.) mechanisms [145, 148–151].

After its secretion, the mature or activated protein dimers process a signal through transmembrane Ser-Thr receptor kinases [144]. The TGF-*β* family of receptors is comprised of type I and type II receptors. Type II receptors selectively bind their respective ligands to define part of the specificity of signal transduction [144]. Ligand binding can either be “sequential” or “cooperative” and may involve an accessory receptor (type III) to enhance ligand presentation [152]. Following receptor-ligand interaction, the type II receptor forms a heterotetrameric complex with the type I receptor to transphosphorylate residues of the Gly-Ser (GS) box [152]. The activated type I receptors then phosphorylate Smad-dependent or Smad-independent substrates to regulate the transcription of target genes [153].

Smad proteins exist in three families: receptor-activated, common mediator, and inhibitory. Receptor-activated (R-) Smads dock onto type I receptors and are phosphorylated on distal serine residues following receptor activation [153]. Phosphorylated R-Smads dissociate from the receptor and interact with common mediator Smad4 [153]. The oligomeric R-Smad/Smad4 complex then translocates to the nucleus where it alters the transcription of target genes [153]. Type I receptors not only function through Smad signaling but may also directly activate Smad-independent pathways such as TGF-*β*-activated kinase 1 (TAK1), Ras, nuclear factor-*κ*B (NF-*κ*B), and the mitogen-activated protein kinase (MAPK) subfamily members [154–159]. The variety of direct and context-dependent downstream signaling pathways preserves the multifunctional role(s) of TGF-*β* superfamily ligands while providing the specificity required to control distinct target genes.

### 8.2. Immune Response

The canonical members of TGF-*β sensu stricto* maintain immunological function by regulating the initiation and resolution of the immune response and a comprehensive review has been previously published [160]. Briefly, activated TGF-*β* at the site of injury may initiate a proinflammatory milieu characterized by matrix remodeling and the recruitment and activation of leukocytes [160, 161]. TGF-*β* may then aid in resolving the primary immune response and support a milieu for tissue repair and immunological memory to progress by suppressing the proliferation, differentiation, and survival of a subset of lymphocytes [160].

To initiate an immune response, TGF-*β* may mobilize monocytes, mast cells, and granulocytes to the site of injury and influence their adhesion to the extracellular matrix [160, 162–164]. While TGF-*β* may also recruit monocyte-derived macrophages, their activation and function are typically inhibited to help resolve the immune response [161, 165, 166]. Since immune cells continue to infiltrate the site of injury, the extracellular matrix undergoes pathological remodeling characterized by protease secretion and matrix degradation [167]. TGF-*β* supports the remediation and repair of these tissues by increasing the deposition of matrix proteins and inhibiting protease activation [168].

To sustain the resolution of the immune response, TGF-*β* may regulate T-cell proliferation, differentiation, and survival [169]. TGF-*β* promotes T-cell growth arrest by suppressing interleukin-2 in areas of subthreshold antigen presentation [160, 170]. During the polarizing conditions of the immune response, TGF-*β* maintains peripheral immunological tolerance by inducing the transcription factor FoxP3 to promote CD4+ CD25+ T-cell differentiation to regulatory T cells [160, 171]. CD4+ T-cell differentiation to the T helper (Th) 1 and Th2 cell lineages, however, is inhibited by TGF-*β* mediated repression of the transcription factors T-bet and GATA-3, respectively [169, 172]. In addition to its effects on CD4+ T cells, TGF-*β* may also attenuate the cytotoxicity of CD8+ T cells by inhibiting its cytolytic genes [173].

TGF-*β* not only stabilizes T-cell expression and function to resolve the immune response but also regulates B-cell proliferation, survival, and development [174]. TGF-*β* inhibits both the proliferation and cell cycle progression of B cells through Smad-dependent or Smad-independent pathways [160, 175–177]. TGF-*β* utilizes comparable B-cell growth arrest pathways, as well as a distinct Smad-independent pathway, to induce the apoptosis of B cells [160, 178]. Lastly, TGF-*β* may regulate the maturation and activation of B cells through its induction of isotype switching, suppression of B-cell antigen receptor signaling, and inhibition of immunoglobulin secretion [160, 179, 180].

### 8.3. Nociception

The members of TGF-*β sensu stricto* contribute to both the peripheral and central processing of noxious stimuli. TGF-*β*1 and TGF-*β*2 have been demonstrated to increase de novo neuropeptide synthesis in the DRG that may directly sensitize primary afferent nociceptors [181, 182]. TGF-*β* may also influence DRG excitability by regulating several ion channels including the voltage-gated potassium (Kv) channel and TRPV-1. Application of recombinant TGF-*β*1* in vitro* has been demonstrated to downregulate KCNA4 gene expression and decrease A-type Kv currents in primary DRG cultures [183]. Additionally, TGF-*β*1 Smad-independent signaling may phosphorylate TRPV-1 on Thr residues and potentiate capsaicin-evoked calcium influx in the DRG [184, 185]. The subsequent prolonged depolarization and an impaired repolarization may lead to an amplification of nociceptive transmission and CNS input.

Unlike its role in the periphery, TGF-*β* in the CNS appears to be neuroprotective by regulating neuronal and nonneuronal response to inflammatory injury [186]. Nonneuronal glial cells have recently been recognized to enhance the proinflammatory milieu and facilitate the central processing of nociception [187]. Activated TGF-*β* in the CNS may inhibit the proliferation and activation of these spinal glial cells to attenuate the induction of neuropathic pain [188–190]. TGF-*β* may further reduce excitatory synaptic transmission of second-order neurons by directly suppressing the proinflammatory milieu in the spinal cord [189]. As a result of its biphasic and modulatory role in the peripheral and central transmission of nociception, TGF-*β* appears to have a profound impact on the perception of pain and may initiate, in part, pathological pain syndromes.

### 8.4. Role(s) in Cystitis

TGF-*β* ligands and its cognate receptors are expressed at low, basal levels in rat urinary bladder tissues [191]. Following chemically (CYP) induced cystitis of varying durations, TGF-*β* ligand, and receptor expression appears to display a time- and tissue-dependent regulation. TGF-*β* exhibits a delayed, but sustained, increase in urinary bladder gene and protein expression 8–48 h after CYP treatment [143, 191, 192]. Furthermore, urinary excretion of active and latent TGF-*β*1 is increased up to 100-fold 24 h after acute CYP treatment [143]. The aforementioned regulation of TGF-*β* gene and protein expression has been suggested to be more pronounced in the afferent limb of the micturition reflex suggesting a possible role in the development of LUT symptoms [191]. Its role in micturition reflex dysfunction was confirmed following the pharmacological inhibition of aberrant TGF-*β* signaling with cystitis. Inhibition of TGF-*β* type I receptors 48 h after CYP-induced cystitis decreased urinary frequency and increased bladder capacity, void volume, and intercontraction intervals [191]. These studies raise the possibility of targeting TGF-*β* at the level of the urinary bladder to alleviate voiding dysfunction with cystitis.

## 9. Perspectives and Future Directions

Blockade of cytokine/receptor and chemokine/receptor signaling may represent a potential therapeutic target for inflammation-associated bladder dysfunction. In addition, the presence of certain inflammatory molecules in patient urine may be useful biomarkers for BPS/IC or other bladder disorders such as overactive bladder (OAB). Similar to BPS/IC, the etiology of OAB remains elusive; however, based on patient biopsies an inflammatory contribution has been suggested [193–195]. Tyagi et al. [196] detected a 10-fold increase of CCL2 and the soluble fraction of the CD40 ligand (CD40L) in the urine of OAB patients versus controls. Various cytokines, epidermal growth factor (EGF), and the oncogene GRO-a were also elevated (3-5-fold) in the urine of OAB patients [196]. Whether certain inflammatory mediator/receptor interactions and downstream signaling pathways are redundant or unique across diverse bladder dysfunction or pelvic pain syndromes remains to be determined. Identification of urinary biomarkers in BPS/IC, OAB, or other bladder dysfunctions would improve diagnostic strategies and reduce invasiveness to the patient, improving exclusionary criteria, reducing time to diagnosis and aid in patient selection for pharmacological trials.

## Abbreviations

BPS: Bladder pain syndrome
CCR2: Chemokine (C-C motif) receptor 2
CGRP: Calcitonin gene-related peptide
CNS: Central nervous system
CYP: Cyclophosphamide
DRG: Dorsal root ganglia
DVR: Decapentaplegic-Vg-related
EGF: Epidermal growth factor
ELISA: Enzyme-linked immunosorbent assay
EPSC: Excitatory postsynaptic current
FIC: Feline interstitial cystitis
GS: Glycine-serine
h: Hour(s)
HIV: Human immunodeficiency virus
IC: Interstitial cystitis
Kv: Voltage-gated potassium
L: Lumbar
LAP: Latency associated peptide
LTBP: Latent transforming growth factor-beta binding protein
LUT: Lower urinary tract
MAPK: Mitogen-activated protein kinase
MCP: Monocyte chemoattractant protein
NF-*κ*B: Nuclear factor-kappa B
NGF: Nerve growth factor
NIDDK: National Institute of Diabetes and Digestive and Kidney Diseases
OAB: Overactive bladder
PAG: Periaqueductal gray
PMC: Pontine micturition center
PNS: Peripheral nervous system
PS: Protamine sulfate
qRT-PCR: Quantitative reverse transcriptase polymerase chain reaction
R-: Receptor-activated
S: Sacral
SP: Substance P
T: Thoracic
TAK1: Transforming growth factor-beta-activated kinase 1
TGF-*β*: Transforming growth factor-beta
Th: T helper
TRP: Transient receptor potential
V: Vanilloid.
